# The impact of home medications on the risk of delayed cerebral ischemia after aneurysmal subarachnoid hemorrhage

**DOI:** 10.1007/s00701-025-06730-1

**Published:** 2025-11-26

**Authors:** Pikria Ketelauri, Meltem Gümüs, Hanah Hadice Karadachi, Anna Michel, Aigerim Togyzbayeva, Laurèl Rauschenbach, Nika Guberina, Cornelius Deuschl, Yan Li, Marvin Darkwah Oppong, Yahya Ahmadipour, Philipp Dammann, Ulrich Sure, Ramazan Jabbarli

**Affiliations:** 1 Department of Neurosurgery and Spine Surgery, University Hospital Essen, Hufelandstr. 55, 45147 Essen, Germany; 2 Department of Radiotherapy, University Hospital Essen, Hufelandstr. 55, 45147 Essen, Germany; 3 Institute for Diagnostic and Interventional Radiology and Neuroradiology, University Hospital Essen, Hufelandstr. 55, 45147 Essen, Germany

**Keywords:** Aneurysmal subarachnoid hemorrhage, Cerebral infarction, Delayed cerebral ischemia, Home medications

## Abstract

**Objective:**

Delayed cerebral ischemia (DCI) is one of the most severe complications following aneurysmal subarachnoid hemorrhage (SAH) and can significantly worsen clinical outcomes. This study aimed to analyze the association between patients’ home medications and the risk of cerebral infarction and poor functional outcomes after SAH.

**Methods:**

This retrospective analysis included 995 patients with aneurysmal SAH treated at our clinic between January 2003 and June 2016. Various demographic and clinical baseline characteristics were examined, with a particular focus on regular use of home medications. The study endpoints were the occurrence of early (within 72 h post-SAH) and DCI-related infarcts (> 72 h) in follow-up computed tomography scans, as well as the functional disability at six months, defined as a modified Rankin Scale > 2.

**Results:**

There was no association between the occurrence of early infarcts and patients’ regular medications. In contrast, individuals with calcium channel blockers (CCB) use (*n* = 93) showed a higher rate of DCI (32.6% vs 19.3%, *p* = 0.005) and 6-months functional disability (57.8% vs 46.8%, *p* = 0.048). In multivariable analysis, CCB use was independently associated with the risk of DCI (adjusted odds ratio [aOR] = 4.05; *p* < 0.0001) and functional disability after six months (aOR = 2.73; *p* = 0.036).

**Conclusions:**

Regular CCB use was independently associated with an increased risk of DCI and functional disability at six months. These findings warrant cautious interpretation and further validation in prospective studies.

**Supplementary Information:**

The online version contains supplementary material available at 10.1007/s00701-025-06730-1.

## Introduction

Delayed cerebral ischemia (DCI) is a severe complication after aneurysmal subarachnoid hemorrhage (SAH), which typically occurs in 20% to 40% of cases between the fourth and fourteenth day after the SAH event [37, 38]. DCI is one of the leading causes of secondary neurological deterioration and has been shown to contribute to increased morbidity and mortality [37, 38]. The occurrence of DCI significantly impacts long-term recovery, as it is associated with a higher risk of permanent disability and cognitive impairment [2, 6, 9, 38]. Therefore, a better understanding of the risk factors for DCI is crucial for optimizing patient outcomes following SAH.

The pathophysiology of DCI is multifactorial and involves mechanisms such as microvascular dysfunction, cortical spreading depolarizations, inflammatory responses, and delayed vasospasm [2, 13, 16, 28]. Although numerous studies have investigated DCI risk factors [2, 6, 19], limited research has specifically examined the role of home medications in influencing DCI risk. Among these, calcium channel blockers (CCBs) have garnered particular interest due to their widespread use in arterial hypertension and other cardiovascular diseases and their established role in SAH management. Nimodipine, a dihydropyridine CCB, remains the only pharmacological intervention proven to improve functional outcomes in SAH patients by reducing the risk of DCI-related infarcts [38]. Apart from Nimodipine, numerous studies have investigated other medications with potential advantages or disadvantages concerning the risk of infarction in patients with SAH including statins [24, 25, 39], magnesium [10, 12, 35], antihypertensive agents [1, 4, 5, 12], and antiplatelet drugs [10, 35]. However, inconsistent and contradictory results have so far prevented their broad clinical implementation. Given this incomplete evidence, there is an urgent need for further research into the potential positive or negative effects of home medications on the development of DCI and clinical outcomes in SAH patients. Cardiovascular medications warrant special attention, as they are widely used, and the current body of evidence on their effects remains contradictory.

The aim of this study was to investigate whether, and which, home medications in patients with aneurysmal SAH are associated with the occurrence of cerebral infarctions. Additionally, it assessed the impact of continued home medication after SAH on patients’ functional outcomes. The findings aim to identify potentially relevant agents that may influence the risk of DCI and poor clinical outcomes, thereby contributing to developing individualized therapeutic strategies, particularly in intensive care settings.

## Materials and methods

### Patient population

Patients with aneurysmal SAH who were treated at our university hospital between January 2003 and June 2016 were included in this retrospective study. All included individuals underwent at least one follow-up imaging evaluation through computed tomography (CT). This cohort study was approved by the Ethics Committee of the Medical Faculty of the University of Duisburg-Essen (registration number 15–6331-BO). The study was registered in the German Clinical Trials Register (DRKS) under the unique identifier DRKS00008749.

### Subarachnoid hemorrhage management

All SAH patients were admitted to our intensive care unit and treated by our internal hospital standards and the current treatment guidelines [7, 29]. In all patients, including those with poor clinical grade, medications—such as oral nimodipine—were administered via naso-gastric tube when oral intake was not possible, following standard ICU protocols. Depending on the availability and quality of prior imaging studies – potentially conducted at referring hospitals – an immediate follow-up CT scan, along with CT angiography or digital subtraction angiography (DSA), was performed upon admission. The therapeutic strategy was determined through an interdisciplinary discussion involving the attending neuroradiologist. Aneurysm occlusion was carried out within 24 h of hospital admission, either surgically via clipping or endovascularly, depending on the aneurysm size and location.

Patients with confirmed acute hydrocephalus or those in neurologically unevaluable conditions received external ventricular drainage (EVD) prior to aneurysm treatment to facilitate continuous intracranial pressure (ICP) monitoring. In cases of persistent ICP elevation > 20 mmHg, escalation of ICP management was pursued, either conservatively or surgically, including decompressive craniectomy when indicated.

### Vasospasm management

Preventive measures for vasospasm management included multiple daily neurological examinations and at least one daily transcranial Doppler (TCD) ultrasound examination (Multi-Dop T, Compumedics Germany GmbH/DWL, Singen, Germany). All patients received nimodipine at a daily dose of 360 mg (divided into 6 × 60 mg doses orally).

Prior to aneurysm occlusion, systolic blood pressure was maintained at < 150 mmHg. After aneurysm treatment, the mean arterial pressure (MAP) was kept at ≥ 70 mmHg using colloids and/or catecholamines (norepinephrine, potentially in combination with dobutamine) to ensure a cerebral perfusion pressure of > 60 mmHg. In patients with a progressive increase in mean flow velocity on TCD > 120 cm/s or angiographically confirmed vasospasms on DSA, MAP was further increased to ≥ 90 mmHg. The indication for DSA in suspected vasospasm was determined through an interdisciplinary discussion with the attending neuroradiologist, based on the patient's clinical condition (unexplained neurological deterioration) and persistent abnormal TCD findings. In cases of confirmed angiographic vasospasm, invasive endovascular treatment was performed, consisting of intra-arterial administration of nimodipine and, if necessary, balloon angioplasty.

### Indications for CT scans

Indications for CT examinations included: post-interventional or post-operative monitoring following aneurysm treatment and/or surgical management of acute hydrocephalus via EVD placement (CT imaging was performed within 24 h in such cases), persistent ICP elevation > 20 mmHg, as well as neurological deterioration or impaired consciousness. DCI was defined on non-contrast CT scans as newly developed hypodense cortical or subcortical areas detectable only from the fourth day after the SAH event. Cerebral infarctions that had already manifested before this period, i.e., by the third day post-SAH, were classified as early cerebral infarctions (ECI). Hypodensities attributable to surgical interventions or intracerebral hemorrhage (ICH) were excluded from infarct classification. All CT scans were stored in the institution's picture archiving and communication system and reviewed by the senior author (R.J.) blinded at that time for any clinical information.

### Data management

The data for this study were retrieved from our institutional database for SAH. Comprehensive data on patients and SAH-associated characteristics were collected and analyzed. The following variables were categorized and included for further investigation:

- Patient demographic parameters: age and sex;
- Regular home medication, defined as medication that had been prescribed prior to the SAH, along with the associated comorbidities. Only those medications that were continued after the hemorrhagic event were considered in the analysis. In particular, cardiac medications such as beta blockers, CCBs, angiotensin-converting enzyme (ACE) inhibitors, and angiotensin II receptor blockers (ARBs) were typically maintained. In contrast, medications such as antiplatelet agents (e.g., aspirin), vitamin K antagonists (e.g., phenprocoumon), and drugs that impair hematopoiesis or wound healing (e.g., cytostatic or chemotherapeutic agents) were usually withheld during the acute phase of SAH. Importantly, the routine administration of nimodipine as part of standard SAH treatment was not classified as CCB use in the context of regular home medication, as it is a protocolized therapeutic intervention initiated after the bleeding event for all SAH patients.
- SAH-associated baseline characteristics and complications, including severity of disease assessed by the World Federation of Neurosurgical Societies scale [36] (WFNS) and the original Fisher score [16], aneurysm rebleeding before treatment, angiographic vasospasm, increased ICP.
- For the evaluation of functional outcomes, we used data from routine clinical assessments of SAH patients conducted at our outpatient service six months post-SAH, following our institutional standard operating procedures. Outcomes were classified according to the modified Rankin Scale (mRS) [35]. Assessment was performed during scheduled outpatient visits or via structured telephone interviews with patients or caregivers. Outcome assessors were independent and blinded to treatment details.

For the subsequent statistical analyses, continuous and categorical variables were dichotomized based on common criteria.

### Study endpoints and statistical analysis

The main objective of this study was to determine the impact of preexisting home medication on the risk of cerebral infarction and poor outcome after SAH. Consequently, the following endpoints were selected for this study: occurrence of ECI and DCI in the follow-up CT scans, in-hospital mortality and functional disability after six months, defined as a mRS > 2.

The relationship between recorded home medication and the above-mentioned endpoints was assessed using univariable and multivariable analyses. First, univariable analyses using the Chi^2^ test, Fisher's exact test, or Kendall's Tau test were performed. Subsequently, those home medications that demonstrated a significant p-value in the univariable analyses were then included in the multivariable binary logistic regression analyses. Relevant confounders such as age (dichotomized at the cohort’s mean age), WFNS (grades IV-V vs I-III) and Fisher (grade III-IV vs I-II) scales upon admission, aneurysm rebleeding, treatment modality, angiographic vasospasm, ICP increase > 20 mmHg, acute hydrocephalus, and comorbidities related to the analyzed home medications (arterial hypertension and cardiac diseases) were considered in the adjustments. Since preexisting CCB use showed a significant association with clinical outcomes in the overall cohort, additional multivariable subgroup analyses were performed according to the absence of major comorbidities (chronic kidney disease (CKD), diabetes mellitus, and smoking status). Analyses within comorbidity subgroups were not conducted due to the very small number of patients per group (< 15 per stratum), which precluded reliable statistical estimation. In evaluating temporal pattern of infarct risk following SAH, Kaplan–Meier survival analysis was additionally conducted to examine the association between specific home medications and the timing of cerebral infarction.

All statistical analyses were conducted using SPSS (Version 25, SPSS Inc., IBM). Data visualizations and graphical representations were created using GraphPad Prism 10. Statistical significance was defined as a *p*-value < 0.05.

## Results

### Population and baseline characteristics of the entire cohort

SAH patients treated at our clinic from January 2003 to June 2016 were enrolled in the present study. A total of 995 cases were included in the final analysis. The main baseline characteristics of the entire cohort collected for the study are listed in Table 1.

**Table 1:** Major baseline characteristics of the patients in the final cohort

Demographic characteristics:	*n* (%*) or mean (± SD)
Number of patients	995
Age, years	54.7 (± 14)
Sex, female	667 (67%)
SAH characteristics & complications:	
WFNS (grade 4–5)	413 (41.5%)
Fisher (grade 3–4)	753 (75.7%)
Aneurysm rebleeding	58 (5.8%)
Treatment modality (clipping)	365 (36.7%)
ICP increase > 20 mmHg	440 (44.2%)
Angiographic vasospasm	331 (33.3%)
Acute hydrocephalus	694 (69.7%)
Regular medication:	
Beta blockers	147 (14.8%)
Anti-inflammatory drugs	62 (6.2%)
CCB	93 (9.3%)
ACE inhibitors	169 (17.0%)
ARBs	55 (5.5%)
Statins	52 (5.2%)
L-Thyroxine	101 (10.2%)
Comorbidities:	
Arterial hypertension	682 (68.5%)
Hypercholesterinemia	80 (8.0%)
Cardiac comorbidity	104 (10.5%)
Thyroid dysfunction	130 (13.1%)

*SD* Standard deviation, *SAH* subarachnoid hemorrhage, *WFNS* World Federation of Neurosurgical Societies, *ICP* Intracranial pressure, *CCB* Calcium channel blocker, *ACE* Angiotensin converting enzyme, *ARBs* Angiotensin II receptor blockers. * – percentages were calculated based on the cases with known value

The following frequences of the major regular home medications continued during SAH were reported in the analyzed cohort: beta blockers – 14.8%, anti-inflammatory drugs – 6.2%, CCBs – 9.3%, ACE inhibitors – 17.0%, ARBs – 5.5%, statins – 5.2%, and L-thyroxine – 10.2%.

### Association between home medication and ECI/DCI

ECI was identified in 345 (34.7%) patients. In the univariable analysis (Table 2), none of the analyzed medications demonstrated significant associations with ECI occurrence.

**Table 2:** Univariable analysis of home medications and comorbidities predicting ECI and DCI

Medication:	ECI	DCI
	aOR (95% CI); *p*-value	aOR (95% CI); *p*-value
Beta blockers	0.85 (0.58—1.25); 0.45	1.40 (0.92—2.12); 0.12
Anti-inflammatory drugs	0.86 (0.49—1.50); 0.68	0.83 (0.43—1.63); 0.74
CCB	1.16 (0.74—1.82); 0.56	2.02 (1.26—3.24); **0.005**
ACE inhibitors	0.72 (0.50—1.04); 0.09	1.35 (0.91—2.00); 0.14
ARBs	1.01 (0.57—1.79);1.00	1.38 (0.74—2.59); 0.30
Statins	1.27 (0.72—2.25); 0.46	0.80 (0.38—1.67); 0.72
L-Thyroxine	1.00 (0.65—1.55); 1.00	0.89 (0.52—1.50); 0.70

*ECI* Early brain injury, *DCI* Delayed cerebral ischemia, *aOR* Adjusted odds ratio, *CI* Confidence interval, *CCB* Calcium channel blocker, *ACE* Angiotensin converting enzyme, *ARBs* Angiotensin II receptor blocker

**Fig. 1:**
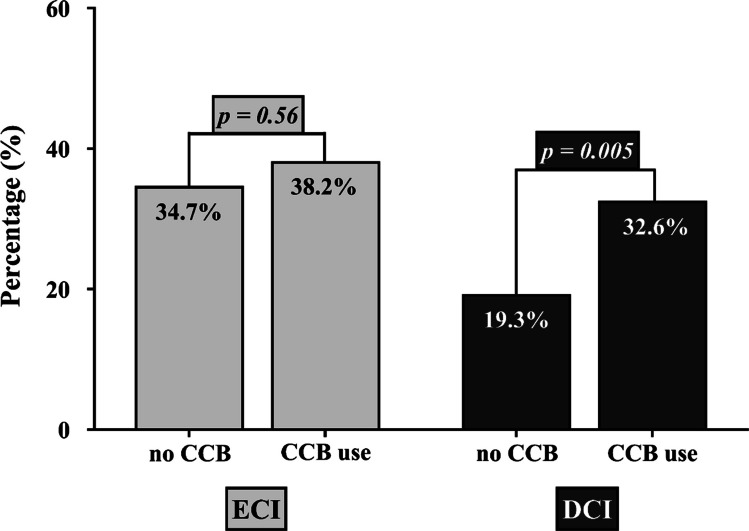
Bar chart showing the percentage distribution of DCI and ECI in groups of patients with and without CCB use. Abbreviations: DCI, delayed cerebral ischemia; ECI, early brain injury; CCB, calcium channel blocker

**Fig. 2:**
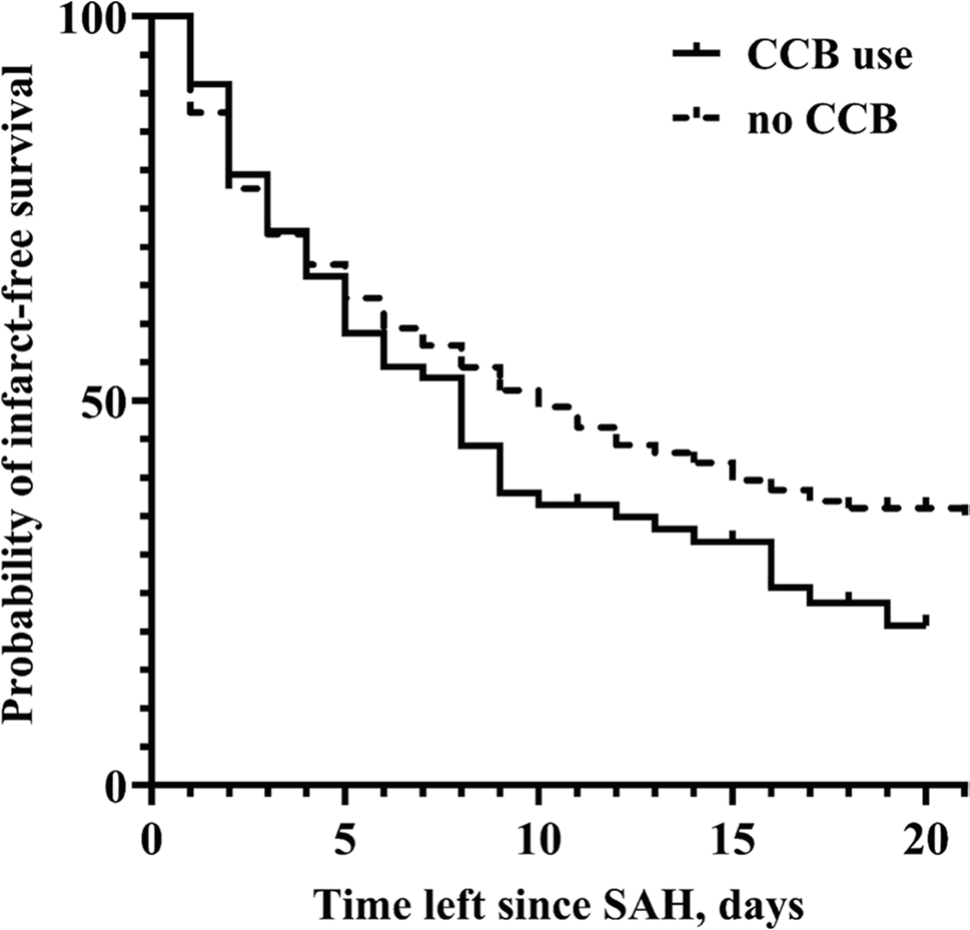
Kaplan–Meier survival plot depicting the probability of infarct-free survival within 3 weeks after SAH in patients with and without preexisting CCB medication. Abbreviations: SAH, subarachnoid hemorrhage; CCB, calcium channel blocker

A total of 205 patients (20.6%) developed DCI during hospitalization. The distribution of home medication among the patients with and without DCI revealed an association between CCB use and the risk of DCI (odds ratio [OR] = 2.02; *p* = 0.005), while other home medications showed no significant correlation (Table 2). The DCI incidence was 19.3% in patients without preexisting CCB use, compared to a significantly higher rate of 32.6% in those on CCBs (Fig. 1). A Kaplan–Meier survival plot illustrates distinct trajectories of infarct-free survival between individuals with and without CCB use (Fig. 2). After adjusting for potential confounders, preexisting CCB use remained an independent predictor of DCI in the final multivariable logistic regression analysis (adjusted OR [aOR] = 4.05; *p* < 0.0001, Table 3).

**Table 3:** Multivariable analysis of the predictors of DCI

Endpoint: DCI	aOR (95% CI)	*p*-value
Age (≥ 55 years)	1.72 (1.04—2.85)	**0.034**
WFNS (grade 4–5)	1.09 (0.66—1.80)	0.75
Fisher (grade 3–4)	1.23 (0.50—3.03)	0.66
Treatment modality (clipping)	0.36 (0.22—0.58)	** < 0.0001**
Angiographic vasospasm	2.66 (1.54—4.62)	** < 0.0001**
Acute hydrocephalus	2.32 (1.02—4.01)	**0.043**
ICP increase > 20 mmHg	2.46 (1.45—4.18)	**0.001**
Aneurysm rebleeding	2.54 (1.01—6.39)	**0.049**
Cardiac comorbidity	0.41 (0.15—1.14)	0.09
Arterial hypertension	0.65 (0.38—1.10)	0.11
CCB (regular medication)	4.05 (1.88—8.73)	** < 0.0001**

*DCI* Delayed cerebral ischemia, *aOR* Adjusted odds ratio, *CI* Confidence interval, *WFNS* World Federation of Neurosurgical Societies, *ICP* Intracranial pressure, *CCB* Calcium channel blocker

**Fig. 3:**
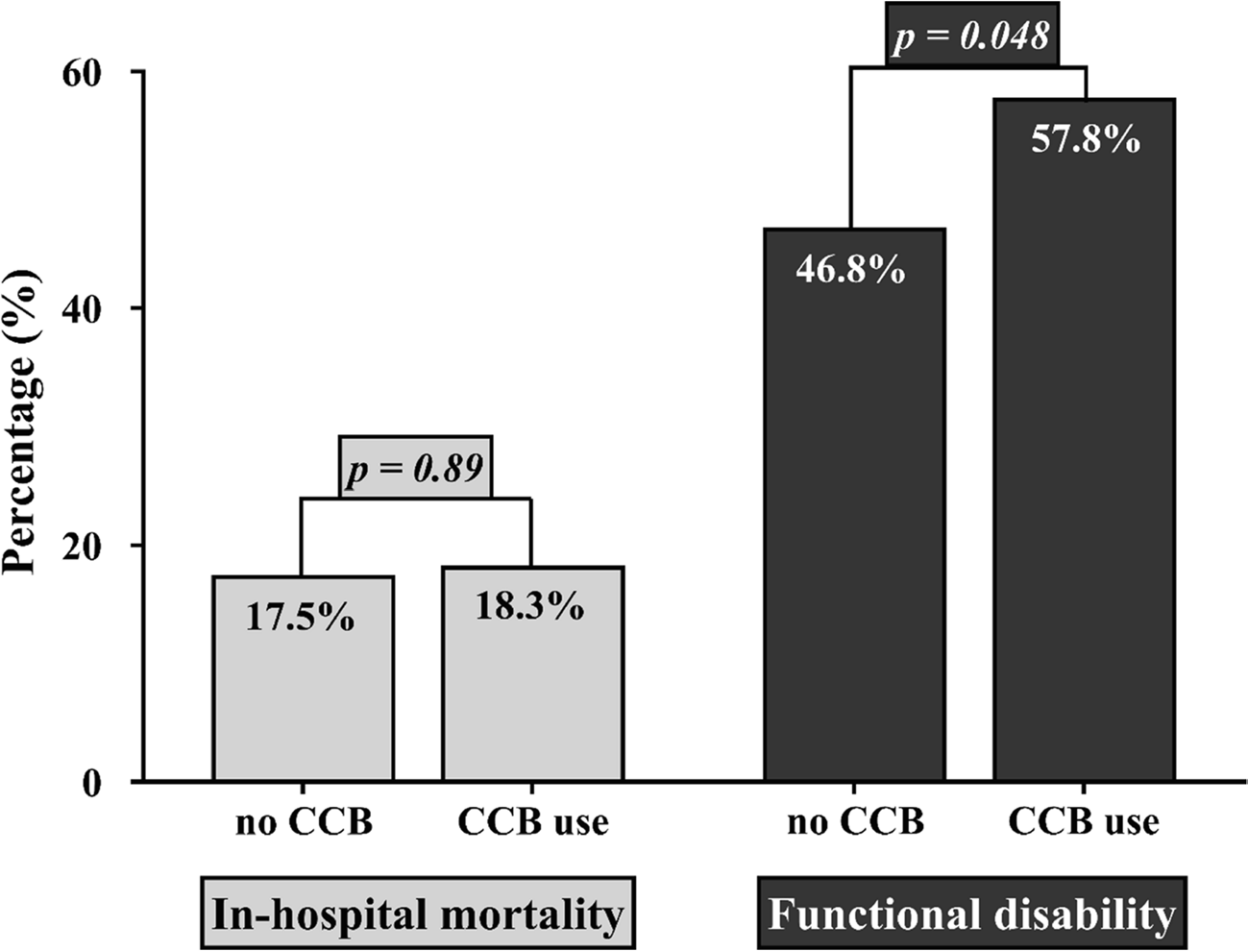
Bar chart showing the percentage distribution of in-hospital mortality and functional disability at six months (mRS > 2) in groups of patients with and without CCB use. Abbreviations: mRS, modified Rankin Scale; CCB, calcium channel blocker

### Association between home medication and functional outcome

Functional disability at six months was observed in 471 (47.3%) patients. There was a significant association between the functional outcome and the use of certain home medications. In patients without CCB use, 46.8% exhibited a poor outcome, compared to 57.8% in those receiving CCB therapy (OR = 1.56, *p* = 0.048, Fig. 3). Additionally, the univariable analysis identified other medications such as beta blockers (OR = 1.52, *p* = 0.023), statins (OR = 2.5, *p* = 0.02), and ACE inhibitors (OR = 1.51, *p* = 0.017) were also associated with the risk of poor functional outcomes at 6 months post-SAH (Table S1). Based on these findings, the subsequent multivariable analysis incorporating the above-mentioned significant home medications (Table 4), showed an independent associations between CCB use and functional disability (aOR = 2.64; *p* = 0.037), but not for the remaining home medications.

**Table 4:** Multivariable analysis of the predictors of functional disability at six months

Endpoint: Functional disability at 6 months	aOR (95% CI)	*p*-value
Age (≥ 55 years)	3.44 (2.03—5.83)	** < 0.0001**
WFNS (grade 4–5)	3.08 (1.90—5.01)	** < 0.0001**
Fisher (grade 3–4)	2.30 (0.99—5.35)	0.053
Treatment modality (clipping)	0.91 (0.56—1.49)	0.71
Angiographic vasospasm	1.81 (1.10—2.97)	**0.019**
Acute hydrocephalus	1.92 (1.08—3.41)	**0.026**
ICP increase > 20 mmHg	5.01 (2.98—8.43)	** < 0.0001**
Aneurysm rebleeding	2.37 (0.74—7.63)	0.15
Hypercholesterinemia	0.42 (0.11—1.61)	0.20
Cardiac comorbidity	1.05 (0.39—2.84)	0.93
Arterial hypertension	1.32 (0.76—2.28)	0.32
CCB (regular medication)	2.64 (1.06—6.58)	**0.037**
Statins (regular medication)	2.19 (0.42—11.52)	0.36
ACE inhibitors (regular medication)	1.42 (0.72 −2.79)	0.31
Beta blockers (regular medication)	1.03 (0.50—2.14)	0.93

*aOR* Adjusted odds ratio, *CI* Confidence interval, *WFNS* World Federation of Neurosurgical Societies, *ICP* Intracranial pressure, *CCB* Calcium channel blocker, *ACE* Angiotensin-converting enzyme

In-hospital mortality was observed in 112 (11.3%) patients. No significant correlation with home medication was found for any of the assessed home medications (Supplementary Table S1, see also Fig. 2 for the association between CCB use and in-hospital mortality).

In additional subgroup analyses restricted to patients without major comorbidities (CKD, diabetes mellitus, and smoking), preexisting CCB use remained independently associated with a higher risk of DCI and functional disability at six months. Due to the small number of cases in subgroups with chronic comorbidities (typically < 15 per group), no reliable statistical analyses of CCB use could be performed in these strata. The results are summarized in Supplementary Table S2.

## Discussion

In this study, we investigated the effect of home medication on the risk of cerebral infarction and poor outcome in patients suffering from aneurysmal SAH. The findings indicate that the continuation of home medication with CCB, in addition to standard nimodipine use, was associated with a higher risk of DCI and poorer long-term functional outcomes. These results emphasize the need for careful reassessment of CCB use in individuals with aneurysmal SAH and further research to validate these associations in larger, prospective studies.

### Pharmacological modulation in SAH

Numerous studies have examined risk factors for cerebral infarction and pharmacological strategies aimed at reducing its incidence and improving neurological outcomes [20, 21]. However, most of this research has focused on post-SAH drug therapy, particularly agents such as aspirin, non-steroidal anti-inflammatory drugs, opioids, dexamethasone, levetiracetam, etc. [10, 32, 33]. In contrast, the potential role of home medications – those taken prior to the hemorrhagic event and continued during SAH – remains largely understudied. Evidence regarding their impact on the risk of cerebral infarction or functional outcomes in patients with aneurysmal SAH is sparse. Recent evidence suggests that certain medications, such as sulfonylureas [12], as well as others [24] including lisinopril, amlodipine, simvastatin, metformin, and tamsulosin, may influence neurological outcomes or risk profiles in patients with aneurysmal SAH. For example, Kenning et al. (2024) reported that recent use of antihypertensive medications like lisinopril and amlodipine was associated with an increased incidence of aneurysmal SAH, whereas current use appeared to confer a protective effect [24]. These findings imply a possible time-dependent relationship between medication use and SAH risk, which may also extend to functional outcomes. While the association between chronic antihypertensive therapy and aneurysm rupture risk has been the subject of several investigations [17, 22, 31], the influence of long-term home medications on SAH-related complications – particularly cerebral infarction and its sequelae – has not been systematically explored. Existing data in this area remains limited and, in many cases, contradictory. In light of this gap, our study aimed to examine the potential impact of commonly used home medications on key clinical outcomes following aneurysmal SAH.

Our results suggest that routine statin use before and after hemorrhagic event does not significantly improve functional outcome or protect against cerebral infarcts – whether ECI or DCI, which is consistent with recent studies questioning the benefit of statins in aneurysmal SAH. While early trials showed promising results [26], subsequent research and meta-analyses have produced inconsistent findings with no clear clinical advantage [24, 39]. Our findings are consistent with studies reporting no clear clinical benefit of statin therapy in SAH patients.

Building on these findings, we also explored the potential effects of other commonly prescribed cardiovascular medications, such as beta blockers and ACE inhibitors, on functional outcomes and cerebral infarction. While statins showed no significant association with cerebral infarction or long-term disability, our analysis revealed preliminary signals regarding other drug classes that warrant further attention.

In our study, we observed potential associations between using beta blockers and ACE inhibitors and functional disability at six months in univariable analysis. However, these associations lost statistical significance in the final multivariate assessment.

Our results are generally consistent with previous reports, which showed that while antihypertensive agents can affect TCD findings, they exhibit no independent association with DCI or long-term functional outcomes [31]. Chalouhi et al. (2016) reported that beta blockers are associated with reduced incidence of cerebral vasospasm [13]. However, it remains unclear whether this translates into improved long-term outcomes. Similarly, although ACE inhibitors have been proposed to offer neuroprotective effects via anti-inflammatory actions and modulation of cerebral autoregulation [4, 5, 18], our study did not confirm these benefits. ACE inhibitors were neither associated with increased DCI risk nor did they provide significant protection against functional impairment.

Given these controversial and inconsistent results, our data highlight the need for further prospective studies to more thoroughly investigate the potentially protective or harmful effects of beta blockers, statins, and ACE inhibitors in the context of SAH.

### Home medication with CCB and association with the risk for DCI

The strongest and most novel observation of our study was the association between continuation of home CCBs and an increased risk of DCI, despite concurrent guideline-based nimodipine therapy. Nimodipine remains the only proven pharmacological agent for reducing DCI-related infarction and improving outcome [17, 38]. The addition of further CCBs, however, may lead to excessive blood pressure reduction, reduced cerebral perfusion pressure, and hemodynamic instability [8, 14, 40]. A systematic review by Alviar et al. (2013) showed that dual CCB therapy lowered systolic blood pressure more than monotherapy [3]. However, American College of Cardiology and the American Heart Association guidelines caution against such combinations due to higher mortality in some groups and generally advise against concurrent use of drugs from the same class because of potential risks without clear benefit [40]. Our findings are consistent with these recommendations and raise the hypothesis that continued home CCB use may adversely affect prognosis. In an analysis of 306 aneurysmal SAH patients, Persad-Paisley et al. (2022) [31] found that CCB use was associated with a lower risk of vasospasm in univariate analysis; however, this association did not remain significant for DCI or poor outcome after adjustment for age and clinical severity in multivariate analysis. In contrast, our larger cohort of 995 patients demonstrated a significant, independent association between continued CCB use and an increased risk of DCI as well as poor 6-month functional outcome. Although our findings suggest that home medication with CCBs could potentially be associated with adverse effects in SAH patients, this observation should be interpreted with caution due to study limitations (see below). The discrepancy with Persad-Paisley et al. (2022) may also be related to their smaller sample size, shorter follow-up period, and institutional practice of discontinuing antihypertensive medication at home after aneurysm clipping.

The broader literature on CCBs in cerebrovascular disease highlights ongoing uncertainties. Observational studies suggested that CCBs may reduce aneurysm growth and rupture risk [27], whereas Mendelian randomization studies indicated a possible genetic association with increased risk of aneurysm and SAH [28]. These divergent results illustrate methodological challenges and limit generalizability, especially across different populations. Importantly, none of these investigations specifically addressed patients with acute SAH under standard nimodipine treatment.

In patients with SAH, maintaining optimal cerebral perfusion is of greatest importance. While nimodipine remains the main pharmacological option for DCI prevention, clinicians should be particularly vigilant when assessing the potential risks of additional CCB therapy. Our analysis indicated an association between pre-admission CCB use and the occurrence of DCI as well as poorer functional outcomes. Although these findings are observational and do not currently warrant changes to guideline-recommended nimodipine therapy, they may provide valuable insight into patient risk stratification. Potential mechanisms could involve differences in cerebral autoregulation or calcium-dependent cellular processes affecting the brain’s response to SAH, though this remains speculative given the retrospective design. Alternative blood pressure-lowering strategies may be preferable to reduce the risk of DCI while preserving adequate cerebral blood flow. However, based on our observational data, no treatment recommendations can be made, and further controlled studies are required to confirm these associations and explore underlying mechanisms.

## Study limitations

While this study provides valuable insights into the association between home medications and the risk of DCI in patients with aneurysmal SAH, several limitations must be acknowledged.

First, the retrospective nature of this study introduces inherent biases, such as potential selection bias and incomplete documentation of patients' medication histories, including variations in CCB types, daily doses, and duration of use. Despite efforts to collect comprehensive data, discrepancies in medical records and variations in patient adherence to prescribed home medications may have impacted the accuracy of the findings. Additionally, residual confounding due to unmeasured cardiovascular or hemodynamic factors cannot be excluded. Moreover, as the study population was derived from a single center, the generalizability of the results to institutions with differing protocols or patient demographics may be limited. Multicenter studies with diverse populations are necessary to validate these findings and improve their external validity. Lastly, the definition of DCI relied on clinical and imaging criteria, but subtle ischemic changes might have gone undetected. Therefore, our findings should be interpreted as associative rather than causal. Despite these limitations, the study identifies critical associations that underscore the need for further research. Prospective, controlled trials with standardized medication protocols and long-term follow-up are needed to clarify the causal relationships and optimize treatment strategies for SAH patients at risk of DCI.

## Conclusion

This study identifies an association between the use of CCB and an increased risk of DCI as well as poorer functional outcomes in patients with aneurysmal SAH. These findings suggest that cautious evaluation of continued CCB therapy following a hemorrhagic event may be warranted. However, further research, particularly experimental and prospective multicenter studies, is essential to clarify causality and refine therapeutic strategies.

## Supplementary Information

Below is the link to the electronic supplementary material.

ESM 1 Supplementary Material 1 (DOCX 22.7 KB)

## Data Availability

The data presented in this study are not publicly available due to institutional data protection policies but are available from the corresponding author upon reasonable request.

## Declarations

### Current state

The manuscript is not under consideration for publication in any other journal.

## Abbreviations

ACE: Angiotensin-converting enzyme
aOR: Adjusted odds ratio
ARBs: Angiotensin II receptor blockers
CI: Confidence interval
CT: Computed tomography
DCI: Delayed cerebral ischemia
DRKS: German Clinical Trial Registry
DSA: Digital subtraction angiography
ECI: Early cerebral infarction
EVD: External ventricular drain
ICH: Intracerebral hemorrhage
ICU: Intensive care unit
ICP: Intracranial pressure
IVH: Intraventricular hemorrhage
MAP: Mean arterial pressure
MRI: Magnetic resonance imaging
mRS: Modified Rankin Scale
OR: Odds ratio
SAH: Subarachnoid hemorrhage
SD: Standard deviation
TCD: Transcranial Doppler
WFNST: World Federation of Neurosurgical Societies

## References

[1] Abbasi MH et al (2025) Angiotensin inhibition reduces the risk of subarachnoid hemorrhage in patients with hypertension. Stroke Vasc Interv Neurol 5(1):e001482 10.1161/SVIN.124.001482 PMC11864572 41608399

[2] Alsbrook DL et al (2023) Pathophysiology of early brain injury and its association with delayed cerebral ischemia in aneurysmal subarachnoid hemorrhage: a review of current literature. J Clin Med. 10.3390/jcm12031015 36769660 10.3390/jcm12031015 PMC9918117

[3] Alviar CL et al (2013) Efficacy and safety of dual calcium channel blockade for the treatment of hypertension: a meta-analysis. Am J Hypertens 26(2):287–297 23382415 10.1093/ajh/hps009

[4] Andone S et al (2022) Neuroprotection in stroke-focus on the renin-angiotensin system: a systematic review. Int J Mol Sci. 10.3390/ijms23073876 35409237 10.3390/ijms23073876 PMC8998496

[5] Annoni F et al (2022) Angiotensin-(1–7) as a potential therapeutic strategy for delayed cerebral ischemia in subarachnoid hemorrhage. Front Immunol. 10.3389/fimmu.2022.841692 35355989 10.3389/fimmu.2022.841692 PMC8959484

[6] Ayling OGS et al (2016) Dissociation of early and delayed cerebral infarction after aneurysmal subarachnoid hemorrhage. Stroke 47(12):2945–2951 27827324 10.1161/STROKEAHA.116.014794

[7] Bederson JB et al (2009) Guidelines for the management of aneurysmal subarachnoid hemorrhage: a statement for healthcare professionals from a special writing group of the Stroke Council, American Heart Association. Stroke 40(3):994–1025 19164800 10.1161/STROKEAHA.108.191395

[8] Brandt L et al (1988) Use of a calcium antagonist in aneurysmal subarachnoid hemorrhage. Ann N Y Acad Sci 522:667–675 3288063 10.1111/j.1749-6632.1988.tb33412.x

[9] Burth S et al (2023) Outcome analysis for patients with subarachnoid hemorrhage and vasospasm including endovascular treatment. Neurol Res Pract 5(1):57 37915071 10.1186/s42466-023-00283-3 PMC10621117

[10] Cagnazzo F et al (2019) Antiplatelet therapy in patients with aneurysmal SAH: impact on delayed cerebral ischemia and clinical outcome. A meta-analysis. AJNR Am J Neuroradiol. 10.3174/ajnr.A6086 31171518 10.3174/ajnr.A6086 PMC7048556

[11] Catapano JS et al (2023) Outcomes in patients with aneurysmal subarachnoid hemorrhage receiving sulfonylureas: a propensity-adjusted analysis. World Neurosurg 176:e400–e407 37236313 10.1016/j.wneu.2023.05.073 PMC11578856

[12] Chalouhi N et al (2016) Beta-blocker therapy and impact on outcome after aneurysmal subarachnoid hemorrhage: a cohort study. J Neurosurg JNS 125(3):730–736 10.3171/2015.7.JNS15956 26799296

[13] Dodd WS et al (2021) Pathophysiology of delayed cerebral ischemia after subarachnoid hemorrhage: a review. J Am Heart Assoc 10(15):e021845 34325514 10.1161/JAHA.121.021845 PMC8475656

[14] Drummond JC (2019) Blood pressure and the brain: how low can you go? Anesth Analg. 10.1213/ANE.0000000000004034 30883421 10.1213/ANE.0000000000004034

[15] Fisher CM, Roberson GH, Ojemann RG (1977) Cerebral vasospasm with ruptured saccular aneurysm–the clinical manifestations. Neurosurgery 1(3):245–248 615969 10.1227/00006123-197711000-00004

[16] Geraghty JR, Testai FD (2017) Delayed cerebral ischemia after subarachnoid hemorrhage: beyond vasospasm and towards a multifactorial pathophysiology. Curr Atheroscler Rep 19(12):50 29063300 10.1007/s11883-017-0690-x

[17] Hoh BL et al (2023) 2023 guideline for the management of patients with aneurysmal subarachnoid hemorrhage: a guideline from the American Heart Association/American Stroke Association. Stroke 54(7):e314–e370 37212182 10.1161/STR.0000000000000436

[18] Honda Y et al (1997) Alacepril, an angiotensin-converting enzyme inhibitor, prevents cerebral vasospasm in subarachnoid hemorrhage model in rats. Methods Find Exp Clin Pharmacol 19(10):699–706 9542720

[19] Jabbarli R et al (2020) Laboratory biomarkers of delayed cerebral ischemia after subarachnoid hemorrhage: a systematic review. Neurosurg Rev 43(3):825–833 30306357 10.1007/s10143-018-1037-y

[20] Jabbarli R et al (2015) Predictors and impact of early cerebral infarction after aneurysmal subarachnoid hemorrhage. Eur J Neurol 22(6):941–947 25708292 10.1111/ene.12686

[21] Jabbarli R et al (2015) Early identification of individuals at high risk for cerebral infarction after aneurysmal subarachnoid hemorrhage: the BEHAVIOR score. J Cereb Blood Flow Metab 35(10):1587–1592 25920954 10.1038/jcbfm.2015.81 PMC4640318

[22] Jabbarli R et al (2022) Regular medication as a risk factor for intracranial aneurysms: a comparative case–control study. Eur Stroke J 8(1):251–258 37021158 10.1177/23969873221129080 PMC10069188

[23] Kanning JP et al (2024) Prescribed drug use and aneurysmal subarachnoid hemorrhage incidence. Neurology 102(12):e209479 38838229 10.1212/WNL.0000000000209479 PMC11226321

[24] Liu J, Chen Q (2015) Effect of statins treatment for patients with aneurysmal subarachnoid hemorrhage: a systematic review and meta-analysis of observational studies and randomized controlled trials. Int J Clin Exp Med 8(5):7198–7208 26221259 PMC4509204

[25] Liu Y-F et al (2016) Drug treatment of cerebral vasospasm after subarachnoid hemorrhage following aneurysms. Chin Neurosurg J 2(1):4

[26] Liu Q et al (2023) Association of calcium channel blockers with lower incidence of intracranial aneurysm rupture and growth in hypertensive patients. J Neurosurg 139(3):651–660 36708539 10.3171/2022.12.JNS222428

[27] Liu H et al (2023) Genetically determined blood pressure, antihypertensive medications, and risk of intracranial aneurysms and aneurysmal subarachnoid hemorrhage: a Mendelian randomization study. Eur Stroke J 9(1):244–250 37800876 10.1177/23969873231204420 PMC10916827

[28] Macdonald RL (2014) Delayed neurological deterioration after subarachnoid haemorrhage. Nat Rev Neurol 10(1):44–58 24323051 10.1038/nrneurol.2013.246

[29] Molyneux AJ, Kerr RS (2002) The future management of subarachnoid haemorrhage. J Neuroradiol 29(2):74–75 12297729

[30] Persad-Paisley EM et al (2022) Association of pre-admission antihypertensive agents and outcomes in aneurysmal subarachnoid hemorrhage. J Clin Neurosci 103:119–123 35868228 10.1016/j.jocn.2022.07.013

[31] Shimizu K et al (2021) Candidate drugs for preventive treatment of unruptured intracranial aneurysms: a cross-sectional study. PLoS ONE 16(2):e0246865 33577580 10.1371/journal.pone.0246865 PMC7880482

[32] Solar P et al (2021) Non-steroidal anti-inflammatory drugs in the pathophysiology of vasospasms and delayed cerebral ischemia following subarachnoid hemorrhage: a critical review. Neurosurg Rev 44(2):649–658 32124117 10.1007/s10143-020-01276-5

[33] Sorrentino ZA et al (2023) Evaluating analgesic medications utilized in the treatment of aneurysmal subarachnoid hemorrhage and association with delayed cerebral ischemia. J Clin Neurosci 115:157–162 37579712 10.1016/j.jocn.2023.07.023

[34] Teasdale GM et al (1988) A universal subarachnoid hemorrhage scale: report of a committee of the World Federation of Neurosurgical Societies. J Neurol Neurosurg Psychiatry 51(11):1457 3236024 10.1136/jnnp.51.11.1457 PMC1032822

[35] van den Bergh WM et al (2006) Randomized controlled trial of acetylsalicylic acid in aneurysmal subarachnoid hemorrhage: the MASH study. Stroke 37(9):2326–2330 16888270 10.1161/01.STR.0000236841.16055.0f

[36] van Swieten JC et al (1988) Interobserver agreement for the assessment of handicap in stroke patients. Stroke 19(5):604–607 3363593 10.1161/01.str.19.5.604

[37] Veldeman M et al (2024) Delayed cerebral infarction after aneurysmal subarachnoid hemorrhage: location, distribution patterns, infarct load, and effect on outcome. Neurology 103(3):e209607 38950352 10.1212/WNL.0000000000209607

[38] Vergouwen MD et al (2010) Definition of delayed cerebral ischemia after aneurysmal subarachnoid hemorrhage as an outcome event in clinical trials and observational studies: proposal of a multidisciplinary research group. Stroke 41(10):2391–2395 20798370 10.1161/STROKEAHA.110.589275

[39] Vergouwen MD et al (2010) Effect of statin treatment on vasospasm, delayed cerebral ischemia, and functional outcome in patients with aneurysmal subarachnoid hemorrhage: a systematic review and meta-analysis update. Stroke 41(1):e47-52 19875741 10.1161/STROKEAHA.109.556332

[40] Whelton PK et al (2018) 2017 ACC/AHA/AAPA/ABC/ACPM/AGS/APhA/ASH/ASPC/NMA/PCNA guideline for the prevention, detection, evaluation, and management of high blood pressure in adults: executive summary: a report of the American College of Cardiology/American Heart Association Task Force on Clinical Practice Guidelines. Hypertension 71(6):1269–1324 29133354 10.1161/HYP.0000000000000066

