# Supplementary material for: The impact of home medications on the risk of delayed cerebral ischemia after aneurysmal subarachnoid hemorrhage
Source: Acta Neurochir (Wien). 2025 Nov 26;167(1):296. doi: 10.1007/s00701-025-06730-1 (PMC12660461; doi:10.1007/s00701-025-06730-1)
Supplement: Supplementary file 1 — Supplementary Material 1 (DOCX 22.7 KB) [file 701_2025_6730_MOESM1_ESM.docx]

**Table S1:** Univariable analysis of home medications predicting in-hospital mortality and functional disability at six months (mRS > 2).

| **Medication:** | **In-hospital mortality** | **Functional disability** |
| --- | --- | --- |
|  | **aOR (95% CI); p-value** | **aOR (95% CI); p-value** |
| Beta blockers | 1.13 (0.72 - 1.76); 0.64 | 1.52 (1.06 - 2.17); **0.023** |
| Chronic use of anti-inflammatory drugs | 0.82 (0.40 - 1.70); 0.73 | 0.60 (0.35 - 1.02); 0.07 |
| CCB | 1.06 (0.61 - 1.84); 0.89 | 1.56 (1.00 - 2.41); **0.048** |
| ACE inhibitors | 0.92 (0.59 - 1.43); 0.82 | 1.51 (1.08 - 2.11); **0.017** |
| ARBs | 0.67 (0.30 - 1.51); 0.46 | 1.19 (0.68 - 2.08); 0.57 |
| Statins | 1.80 (0.95 - 3.39); 0.09 | 2.50 (1.37 - 4.58); **0.02** |
| L-Thyroxine | 0.84 (0.48 - 1.47); 0.59 | 0.94 (0.62 - 1.42); 0.83 |

**Abbreviations:** mRS, modified Rankin Scale; aOR, adjusted odds ratio; CI, confidence interval; CCB, calcium channel blockers; ACE, angiotensin converting enzyme; ARBs, angiotensin II receptor blockers.

**Table S2:** Multivariable subgroup analyses of preexisting CCB use and the risk of DCI and Functional disability at 6 months after aneurysmal SAH, stratified by the absence of major comorbidities (CKD, diabetes mellitus, and smoking status).

|  | **DCI**  **aOR (95% CI), p-value** | **Functional disability at 6 months aOR (95% CI), p-value** |
| --- | --- | --- |
|  | **Non-CKD** | |
| Age (≥55 years) | 1.67 (1.01 - 2.78); **0.05** | 3.43 (2.03 - 5.79); **< 0.001** |
| Cardiac comorbidity | 0.36 (0.12 - 1.07); 0.07 | 1.19 (0.43 - 3.30); 0.74 |
| Arterial hypertension | 0.64 (0.38 - 1.09); 0.10 | 1.40 (0.83 - 2.36); 0.21 |
| WFNS (grade 4-5) | 1.05 (0.64 - 1.73); 0.85 | 3.10 (1.90 - 5.04); **< 0.001** |
| Fisher (grade 3-4) | 1.27 (0.51 - 3.13); 0.61 | 2.29 (0.98 - 5.35); 0.06 |
| Treatment modality (clipping) | 0.38 (0.23 - 0.62); **< 0.001** | 0.94 (0.57 - 1.54); 0.79 |
| Acute hydrocephalus | 1.93 (0.97 - 3.83); 0.06 | 1.82 (1.02 - 3.24); **0.04** |
| Aneurysm rebleeding | 2.61 (1.04 - 6.58); **0.04** | 2.52 (0.79 - 7.98); 0.12 |
| Angiographic vasospasm | 2.58 (1.49 - 4.49); **0.001** | 1.86 (1.13 - 3.07); **0.02** |
| ICP increase >20 mmHg | 2.35 (1.39 - 3.99); **0.002** | 4.77 (2.85 - 7.99); **< 0.001** |
| CCB (regular medication) | 4.23 (1.95 - 9.17); **< 0.001** | 3.84 (1.55 - 9.54); **0.004** |
|  | **Non-Diabetes** | |
| Age (≥55 years) | 1.94 (1.15 - 3.28); **0.013** | 3.34 (1.95 - 5.70); **< 0.001** |
| Cardiac comorbidity | 0.41 (0.14 - 1.20); 0.10 | 0.83 (0.30 - 2.34); 0.73 |
| Arterial hypertension | 0.66 (0.38 - 1.14); 0.14 | 1.30 (0.77 - 2.21); 0.33 |
| WFNS (grade 4-5) | 1.09 (0.66 - 1.83); 0.73 | 3.07 (1.88 - 4.99); **< 0.001** |
| Fisher (grade 3-4) | 1.15 (0.46 - 2.88); 0.77 | 2.91 (1.18 - 7.19); **0.021** |
| Treatment modality (clipping) | 0.36 (0.22 - 0.61); **< 0.001** | 0.89 (0.54 - 1.47); 0.64 |
| Acute hydrocephalus | 2.00 (0.99 - 4.07); 0.055 | 2.05 (1.14 - 3.68); **0.016** |
| Aneurysm rebleeding | 3.33 (1.25 - 8.84); **0.016** | 2.57 (0.81 - 8.16); 0.11 |
| Angiographic vasospasm | 3.20 (1.79 - 5.72); **0.001** | 1.91 (1.15 - 3.15); **0.012** |
| ICP increase >20 mmHg | 2.53 (1.47 - 4.35); **0.001** | 4.68 (2.77 - 7.89); **< 0.001** |
| CCB (regular medication) | 3.89 (1.77 - 8.59); **0.001** | 3.20 (1.34 - 7.65); **0.009** |
|  | **Non-Smokers** | |
| Age (≥55 years) | 1.67 (0.93 - 2.97); 0.08 | 3.54 (1.84 - 6.80); **< 0.001** |
| Cardiac comorbidity | 0.53 (0.18 - 1.57); 0.25 | 1.94 (0.54 - 0.54); 0.31 |
| Arterial hypertension | 0.80 (0.44 - 1.46); 0.47 | 1.39 (0.75 - 2.59); 0.30 |
| WFNS (grade 4-5) | 1.00 (0.56 - 1.77); 1.00 | 3.71 (2.05 - 6.72); **< 0.001** |
| Fisher (grade 3-4) | 0.99 (0.35 - 2.85); 0.99 | 1.49 (0.54 - 4.08); 0.44 |
| Treatment modality (clipping) | 0.37 (0.21 - 0.66); **0.001** | 1.00 (0.55 - 1.84); 0.99 |
| Acute hydrocephalus | 1.74 (0.80 - 3.82); 0.16 | 1.35 (0.66 - 2.77); 0.41 |
| Aneurysm rebleeding | 1.91 (0.63 - 5.76); 0.25 | 2.88 (0.57 - 14.43); 0.20 |
| Angiographic vasospasm | 2.73 (1.46 - 5.08); **0.002** | 1.80 (0.98 - 3.29); 0.06 |
| ICP increase >20 mmHg | 2.29 (1.25 - 4.21); **0.008** | 5.93 (3.13 - 11.26); **< 0.001** |
| CCB (regular medication) | 3.24 (1.32 - 7.95); **0.010** | 1.59 (0.60 - 4.21); 0.35 |

**Note**: Subgroup analyses of preexisting CCB use were conducted only in patients without major comorbidities (CKD, diabetes, or smoking). Analyses within comorbidity subgroups were not performed due to the small patient numbers (< 15 per group), which precluded meaningful statistical evaluation.

**Abbreviations:** DCI, delayed cerebral ischemia; aOR, adjusted odds ratio; CI, confidence interval; WFNS, World Federation of Neurosurgical Societies; ICP, intracranial pressure; CCB, calcium channel blocker; CKD, chronic kidney disease.
